# Antitumorigenic Effect of Cannabidiol in Lung Cancer: What Do We Know So Far?–A Mini Review

**DOI:** 10.52547/ibj.3732

**Published:** 2022-10-08

**Authors:** Issaris Vasileios, Gerasimos Panagiotis Milas, Nicholas Zareifopoulos

**Affiliations:** 1 Athens *General Hospital* '*Evangelismos*, Athens, Greece;; 2 Penteli Children’s Hospital, Athens, Greece;; 3 *General Hospital* of *Nikaia* Agios Panteleimon, Athens, Greece

**Keywords:** Cannabidiol, Cannabinoids, Lung cancer

## Abstract

Lung cancer remains a major factor contributing to morbidity and mortality worldwide. CBD and THC could serve as a specific treatment for lung cancer, owing to their essential role in lung cancer cell apoptosis. This review evaluated the antitumorigenic mechanisms of CBD in lung cancer cells. We searched the databases MEDLINE, clinicaltrials.gov, Cochrane Central Register of Controlled Trials, and Google Scholar using specific terms. Of 246 studies screened, nine were included and assessed using the ToxRTool. All the selected studies were conducted *in vitro*, and four of which also had an *in vivo* content. The most common cell line used in all the studies was A549; however, some studies contained other cell lines, including H460 and H358. Our findings suggested that CBD has direct antineoplastic effects on lung cancer cells through various mechanisms mediated by cannabinoid receptors or independent of these receptors. All studies were referred to an *in vitro* model; hence, further research in animals is required.

## INTRODUCTION

**Rationale**

Cancer is a condition characterized by uncontrolled cell growth and acquisition of metastatic properties. As of 2021, lung cancer has been a major public health problem worldwide and is among the leading cause of death in the United States^[^^1^^]^. Smoking tobacco is considered the strongest risk factor for lung cancer, albeit weaker association has been noted for cannabis smoking^[^^2^^,^^3^^]^. SCLC and NSCLC are two main types of lung cancer. SCLC is divided into two different subtypes: small cell carcinoma and mixed small cell/large cell cancer or combined small cell lung cancer. NSCLC is categorized into three types: adenocarcinoma, squamous cell carcinoma, and large cell carcinoma^[^^4^^]^. 

The current treatment for stage I-II of NSCLC includes surgery using video-assisted techniques^[^^5^^]^ together with adjuvant chemotherapy^[^^6^^]^. The current chemotherapeutic guidelines include the use of cisplatin or carboplatin as the main agent combined with a second drug such as etoposide or paclitaxel^[^^7^^]^, as well as the possible use of immunotherapy. For patients with locally advanced NSCLC (stage IIIA-B) who cannot undergo surgical resection of the tumor, the recommended standard of care entails the combination of radiotherapy with duplet chemo-therapy^[^^6^^]^. Patients with NSCLC in stage IV receive individualized therapy, which is performed based on their performance status. These patients are tested for commonly known mutations such as KRAS, EGFR, ALK, ROS1, BRAF, RET, MET, or NTRK*,* which lead to neoplasia, and their first-line treatment regimen includes immunotherapeutic drugs targeting these genes^[^^8^^]^. Patients in the final stage of lung cancer also receive palliative care, which helps them deal with symptoms such as pain, dyspnoea, anxiety, and depression, and improves their quality of life^[^^9^^]^. 

The endocannabinoid system, a biological system in humans, is composed of cannabinoids and their receptors. Anandamide and 2-arachidonoylglycerol are eicosanoid endocannabinoids acting as cannabinoid receptor agonists^[^^10^^]^. CB1 and CB2, the two types of cannabinoid receptors, belong to Gi/o-protein-coupled receptor family and are abundant in the central nervous system. CB1 receptors are mainly found in several brain regions such as the forebrain and hippocampus, and CB2 are expressed not only in glioma cells but also in circulating immune cells and macrophage-derived cells where their expression is greater^[^^11^^]^. Furthermore, exogenous cannabinoids, THC and cannabidiol CBD, isolated from Cannabis sativa, have a similar action as endogenous cannabinoids^[^^12^^]^. Nowadays, THC and CBD are used for palliative care in patients with terminal stage of lung cancer. Moreover, these two compounds can help the patients suffering from chemotherapy side effects such as anorexia, vomiting, depression, and pain^[^^13^^]^. Apart from the use in palliative care, THC and CBD have shown antitumorigenic effects in several cancer cell lines^[^^14^^]^. In contrast to THC, CBD is a safer alternative due to its minimal psychoactive effect, which extensive research has corroborated its safety^[^^15^^]^. 

Given that the effect of CBD as an antineoplastic compound on a molecular level has strongly been supported by many preclinical studies, we aimed to explore the possible mechanism of action of CBD. The data of the current review could assist physicians in understanding the molecular antitumorigenic mechanisms of CBD on lung cancer cells.

## MATERIALS AND METHODS

**Search strategy**

Our review was performed in accordance with the PRISMA guidelines. The PRISMA checklist is presented in the Supplementary Table 1. All studies evaluating the potential role of CDB on specific lung cancer cell lines were deemed eligible for inclusion. Two blinded researchers (V.I. and N.Z.) performed the study selection process in three consecutive steps. At first, titles and abstracts of all found electronic articles were screened. The next step included the full text assessment of articles meeting the inclusion criteria. In the final step, studies reporting the outcome of our interest were included in our review. Any disagreement between the researchers regarding the included articles was resolved by the consensus of all authors. We extracted the following data from the included studies: name of the first author, year of publication, substance used, country where the study was conducted, study design, cell line used, and outcome found.

**Inclusion/exclusion criteria**

**Fig. 1:**
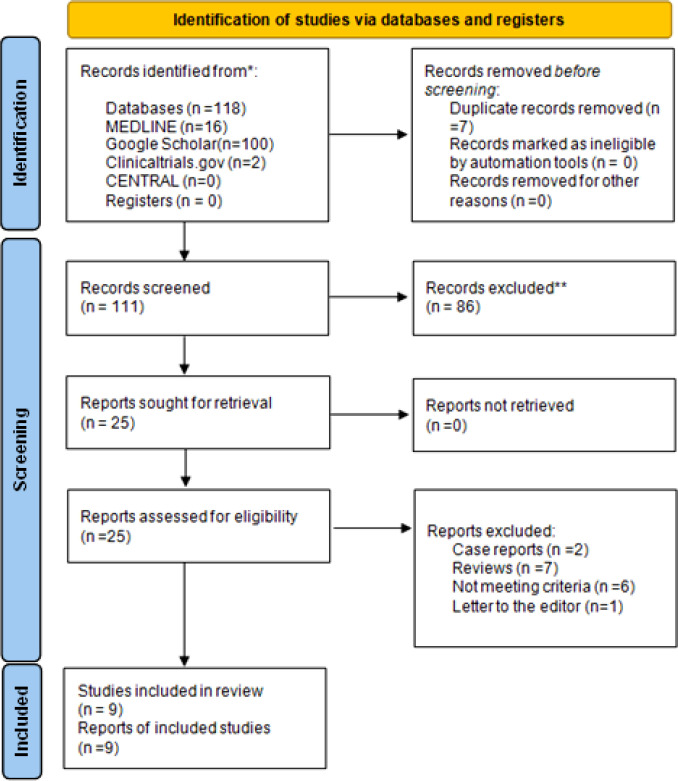
Flow diagram of the literature search

Our literature search was conducted using the terms ("cannabidiol") AND ("lung cancer") in the MEDLINE (1946-2021), Cochrane Central Register of Controlled Trials (CENTRAL; 1999-2021) and Clinicaltrials.gov (2008-2021) databases. The grey literature was assessed by screening the first ten pages of Google Scholar. If necessary, the selected terms were altered, and we used critical search terms depending on the database we searched. We also screened citations found within the included articles (snowball method). No language or date restrictions were applied. The date of the last search was the 27^th^ of October of 2021. The flow diagram of the literature search is presented in Figure 1. We excluded studies reporting outcomes from THC exposure, marijuana smoking, CBD smoking or vaping, and the use of CBD in palliative care. 

**Quality assessment**

The ToxRTool was used to evaluate the quality of the included studies. The tool was initially conceived to assess the risk of bias in toxicological data, but it has found applications in other domains of preclinical research, as well. According to this tool, studies were allotted to Klimisch categories 1, 2, or 3 based on certain defined criteria^[^^16^^]^. We presented our assessment of the included studies in two separate tables, one for *in vitro* (Table 1) and one for *in vivo* studies (Table 2). Criteria were answered with ‘yes’ (score 1) or ‘no’ (score 0).

## RESULTS

A total of 246 studies were selected for inclusion in our review. After the removal of duplicates, 16 studies were included following the full text assessment. Two case reports^[^^17^^,^^18^^]^ and seven review studies^[^^14^^,^^15^^,^^19^^-^^23^^]^ were excluded. Six studies were also excluded due to not meeting the inclusion criteria^[^^20^^,^^24^^-^^28^^]^. One study was a letter to the editor^[^^29^^]^. Overall, this review included nine studies^[^^30^^-^^38^^]^, which met the inclusion criteria we set. The study selection process is shown in Figure 1. All included *in vitro *studies were assigned to Klimisch category 1. Eight out of nine studies received maximum score (18 points) as all criteria were met^[^^30^^-^^33^^,^^35^^-^^38^^]^. One study gained 16 points as it missed criteria 2 and 12 (purity of the test substance and positive controls included)^[^^34^^]^. All four *in vivo* studies^[^^30^^,^^31^^,^^36^^,^^37^^]^ were assigned to Klimisch category 1. Three studies^[^^30^^,^^36^^,^^37^^]^ scored 19 out of 21 due to receiving zero points in questions 6, 8, and 20 (sex, body weight of the studied organisms, and the study design). Choi *et al.*^[^^34^^]^ conducted a study on the effects of CBD in the A549 lung cancer cell line. The authors found a time- and concentration-dependent proapoptotic effect of CBD on A549 cells, which was mediated primarily by the activation of caspases 3, 8 and 9. Exposure to CBD also increased lactate dehydrogenase activity in culture media in a dose-dependent manner, suggesting that this compound may also have a direct cytolytic effect. The majority of the included studies were conducted by a research group in the University of Rostock, Germany^[^^30^^-^^32^^,^^35^^-^^38^^]^,. The invasiveness of A549 cells decreased after exposure to CBD, likely due to the upregulation of TIMP-1, an inhibitor of proteases, which enables tumor cells to invade basement membranes and small vessel endothelial cells. This effect was inhibited by CB and TRPV1 receptor antagonists, suggesting that the effect of CBD is mediated through cannabinoid receptor signaling. CBD did not induce significant cytotoxicity in the A549 cell line, neither *in vitro* nor *in vivo*^[^^30^^]^. The same research group subsequently found that CBD downregulates the expression of PAI-1, a plasminogen system inhibitor that promotes tumor growth, angiogenesis, and invasiveness^[^^37^^]^. This effect was abolished in the presence of cannabinoid or vanilloid receptor antagonists, as observed in the previous study of Ramer *et al.*^[^^30^^]^. To exclude the possibility that this action of CBD is found only in A549 cells, the authors confirmed that CBD exerts the same effect in H460 cells (a large-cell carcinoma cell line) and H358 cells. Treatment with CBD also reduced PAI-I secretion and metastatic foci in athymic nude mice. Further research by the same group investigated the role of ICAM-1 in the effect of cannabinoids on lung cancer cell lines A549, H460, and H358^[^^36^^]^. A concentration-dependent upregulation of ICAM-1 was observed within hours of exposure to CBD, THC and methanandamide, whereas TIMP-1 upregulation occurred within a matter of days. As TIMP-1 upregulation is prevented by ICAM-1 neutralizing antibodies or silencing microRNA, the authors concluded that the cellular adhesion and signaling molecule ICAM-1 are implicated in the beneficial effects of cannabinoid compounds on lung cancer. In addition, the group confirmed that cannabinoids inhibit angiogenesis and neovascularization of tumor cells *in vitro *by the same mechanism, as they prevented migration and tubing of human umbilical vein endothelial cells cultured in the same media as the tumor cell line^[^^35^^]^. In a follow-up study, Ramer *et al.*^[^^31^^]^ examined the role of COX-2 and PPAR-γ in the antitumorigenic and proapoptotic effects of CBD. The exposure of A549 and H460 cells to 3-μM concentration of CBD resulted in the immediate upregulation of COX-2 activity, as well as the upregulation of PPAR-γ expression after a few hours. Activation of the intracellular receptor PPAR-γ by prostaglandin PGD2 gave rise to apoptotic cell death. A particularly important observation is that cannabinoid and vanilloid receptor antagonists did not attenuate the proapoptotic effect of CBD. CBD also upregulates COX-2 expression in the same cell line, although the mechanism for this effect remains unclear. Another research group examined the role of innate immunity in the cytotoxic effects of cannabinoids in lung cancer cell lines. To this end, they cultured A549 and H460 cells and also cells isolated from the brain metastasis of a single lung cancer patient in the media inoculated with natural killer cells isolated from healthy donors^[^^32^^]^. It was observed that cannabinoids induced ICAM-1 expression, resulting in the activation of lymphokine-activated killer cells against the cancer cell lines. This effect was mediated by cannabinoid receptors but could be prevented by ICAM-1 neutralizing antibodies. It must be noted that ICAM-1 expression and the action of lymphokine-activated killer cell was not observed in BEAS-2B cells, an immortalized but non-cancerous bronchial cell line. An independent study utilizing A549, H1792, H460 cell lines found that both CBD and THC reduced the expression of EGFR by a mechanism mediated by the cannabinoid receptors^[^^38^^]^. Combined exposure to THC and CBD resulted in a greater effect than either agent used alone. Due to this mechanism, cannabinoids inhibited epithelial to mesenchymal transition of the cancerous cells, prevented cell migration and restored an epithelial phenotype. In a cohort of 157 lung cancer patients, the increased cannabinoid receptor expression in the primary tumor was associated with prolonged survival^[^^38^^]^. In a recent study included in this review, the authors observed that CBD induced apoptosis in A549 cells, which express p53 in a dose-dependent manner^[^^33^^]^. The effect was much less pronounced in H1299 cell line, which did not express p53, and no dose-response relationship was observed. CBD did not appear to cause necrosis or other nonapoptotic forms of cell death in any meaningful extent.

**Table 1:** Quality assessment *in vitro*

**Study **	**1**	**2**	**3**	**4**	**5**	**6**	**7**	**8**	**9**	**10**	**11**	**12**	**13**	**14**	**15**	**16**	**17**	**18**	**total**	**Klimisch** **category**
Choi *et al.*^[34]^	1	0	1	1	1	1	1	1	1	1	1	0	1	1	1	1	1	1	16	1
Ramer *et al.*^[30]^	1	1	1	1	1	1	1	1	1	1	1	1	1	1	1	1	1	1	18	1
Ramer *et al.*^[31]^	1	1	1	1	1	1	1	1	1	1	1	1	1	1	1	1	1	1	18	1
Haustein *et al.*^[32]^	1	1	1	1	1	1	1	1	1	1	1	1	1	1	1	1	1	1	18	1
Todorova *et al.*^[33]^	1	1	1	1	1	1	1	1	1	1	1	1	1	1	1	1	1	1	18	1
Ramer *et al.*^[35]^	1	1	1	1	1	1	1	1	1	1	1	1	1	1	1	1	1	1	18	1
Ramer *et al.*^[36]^	1	1	1	1	1	1	1	1	1	1	1	1	1	1	1	1	1	1	18	1
Ramer *et al.*^[37]^	1	1	1	1	1	1	1	1	1	1	1	1	1	1	1	1	1	1	18	1
Milian *et al.*^[38]^	1	1	1	1	1	1	1	1	1	1	1	1	1	1	1	1	1	1	18	1

**Table 2:** Quality assessment *in vivo*

**Study**	**1**	**2**	**3**	**4**	**5**	**6**	**7**	**8**	**9**	**10**	**11**	**12**	**13**	**14**	**15**	**16**	**17**	**18**	**19**	**20**	**21**	**total**	**Klimisch category**
Ramer *et al.*^[^^30^^]^	1	1	1	1	1	0	1	0	1	1	1	1	1	1	1	1	1	1	1	0	1	19	1
Ramer *et al.*^[^^36^^]^	1	1	1	1	1	0	1	0	1	1	1	1	1	1	1	1	1	1	1	0	1	19	1
Ramer *et al.*^[^^37^^]^	1	1	1	1	1	0	1	0	1	1	1	1	1	1	1	1	1	1	1	0	1	19	1
Ramer *et al.*^[^^31^^]^	1	1	1	1	1	1	1	0	1	1	1	1	1	1	1	1	1	1	1	0	1	20	1

**Table 3:** Study characteristics

**Author and year**	**Substance used**	**Country of study**	**Study design**	**Cell line**	**Outcome**
Ramer *et al.*^[^^30^^]^	CBD	Germany	*In vitro *and* in vivo*	A549	CBD reduces the invasiveness of A549 cells, this effect is mediated by cannabinoid receptors and the TIMP-1 protein; CBD did not induce significant cytotoxicity in A549 cell line.
					
Ramer *et al.*^[^^31^^]^	CBD	Germany	*In vitro *and* in vivo*	A549, H460, cancer cells isolated from metastatic brain tumor	CBD induces apoptosis in lung cancer cells by a mechanism independent of cannabinoid receptors mediated by PPAR-γ signaling and COX-2 activity.
					
Haustein *et al.*^[^^32^^]^	CBD, THC, R(+)-methanandamide	Germany	*In vitro*	A549, H460 Brain metastasis cells isolated from a single patient	CBD induces ICAM-1 expression in lung cancer cells but not in non-cancerous cells. Increased ICAM-1 expression is mediated by cannabinoid receptors and as a result, cells are more vulnerable to natural killer cell-mediated cytotoxicity; the other cannabinoids tested also increased the expression of ICAM-1 but to a lesser degree of CBD.
					
Todorova *et al.*^[^^33^^]^	CBD	Bulgaria	*In vitro*	A549, H1299	CBD induces apoptosis in A549 cells which express p53 in a dose dependant manner; The effect is much less pronounced in H1299 cell line which did not express p53 and no dose response relationship was observed.
					
Choi *et al.*^[^^34^^]^	CBD	South Korea	*In vitro*	A549	CBD induces tumor cell death in both time- and dose-dependent manner; CBD induces lactate dehydrogenase dose dependant increase from tumor cells; the underlying mechanism of time dependent CBD cytotoxicity is probably due to the activation of caspases 3, 8, and 9, which induce apoptosis.
					
Ramer *et al.*^[^^35^^]^	CBD, THC, and R (+)-methanandamide	Germany	*In vitro*	A549, H460, H358	Cannabinoids reduce cancer cell migration potential by inhibiting angiogenesis via TIMP-1 regulation. This effect is mediated by cannabinoid receptors or TRPV-1.
					
Ramer *et al.*^[^^36^^]^	CBD, THC and R (+)- ethanandamide	Germany	*In vitro *and* in vivo*	A549, H460, H358	CBD and other cannabinoids induce immediate upregulation of ICAM-1 and delay upregulation of TIMP-1; in an *in vivo* model CBD prevented the formation of metastatic nodules and upregulated ICAM-1 and TIMP-1 expression.
					
Ramer *et al.*^[^^37^^]^	CBD	Germany	*In vitro *and* in vivo*	A549, H460, H358	CBD reduces the invasiveness of A549 cells by reducing PAΙ-I in a concentration depending manner; treatment with CBD resulted in a reduced tumor size and low PAΙ-I levels in A549 xenografts in athymic mice.
					
Milian *et al.*^[^^38^^]^	CBD and THC	Spain	*In vitro*	A549, H1792, H460, cancer cells isolated from 157 patients.	CBD and THC both inhibit cell migration and epithelial to mesenchymal transition; combined exposure to CBD and THC resulted in a greater effect than either substance used alone.

## DISCUSSION

Lung cancer is among the leading causes of cancer mortality and morbidity worldwide. Apart from the broad-spectrum chemotherapeutic regiments based on platinum or microtubule inhibition, a number of molecular-based therapeutic strategies have emerged for different types of lung cancer in recent years. Current advances in molecular biology and physiology have shed light on the role of the endocannabinoid system in the pathogenesis of cancer. 

Cannabinoids are now being utilized as a palliative treatment of chemotherapy-induced nausea, vomiting and cancer-related cachexia. In this context, numerous independent groups of researchers have attempted to evaluate the antineoplastic properties of cannabinoids. In this review, we strived to summarize the preliminary experimental data regarding the antineoplastic properties of cannabinoids, which could be beneficial for clinicians. Studies included in this review showed that CBD is active against lung cancer cells by a variety of different mechanisms. CBD may be directly toxic to tumor cells, and it induces apoptosis through caspase activation. This process may be mediated by PPAR-γ and COX-2 upregulation, independent of cannabinoid receptors. In this study, we found two case reports^[^^17^^,^^18^^]^ of lung cancer patients whose tumor responded dramatically to CBD, a finding which is supported by the *in vitro* evidence summarized in our review. A functional p53 gene is required for the proapoptotic action of CBD. Furthermore, CBD increases the expression of TIMP-1 and decreases the expression of PAΙ-I, thereby reducing the invasiveness and metastatic potential of lung cancer cells. By upregulating ICAM-1, cannabinoids render tumor cells vulnerable to the immune system. All these actions of CBD are mediated through the cannabinoid receptors and are abolished in the presence of the cannabinoid receptor antagonists. Importantly, antineoplastic effect of CBD in combination with THC may be more potent than that of either agent used alone. It is evident that CBD has antitumorigenic properties in lung cancer cells, and clinical studies are required to validate these preliminary findings. In this regard, the first step would be to evaluate the *in vitro* experiments presented in this review in animal models of lung cancer^[^^30^^,^^31^^,^^36^^,^^37^^]^. The expression of cannabinoid receptors could be a prognostic factor in lung cancer^[^^38^^]^ and also a predictive factor for response to CBD and/or THC. Moreover, P53 may similarly be a predictive factor for response to CBD, but further studies using different cell lines not expressing p53 (as negative control) are required to test this hypothesis. As smoking is the primary risk factor for lung cancer, smoking cannabis products or cannabis alone with tobacco is definitely not recommended. Further *in vivo* and perhaps clinical studies using other routes of administration of CBD are needed to assess the potential role of CBD in treatment of lung cancer. 

Overall, our review evaluates numerous molecular mechanisms by which CBD could affect lung cancer cells. Most of the studies included in our review employed CBD as monotherapy with THC, and other cannabinoids were used as controls. The included studies had a minimal risk of bias and demonstrated the antitumorigenic effects of CBD *in vitro*. As the experiment conducted in the studies were not homogenous, the results could not be combined in a meta-analysis. Oral cannabinoids have been approved for the palliative treatment of chemotherapy-induced nausea and vomiting. They also attenuate cancer-related cachexia and may exert direct antineoplastic effects on lung cancer cells by a variety of different mechanisms, as demonstrated *in vitro*. Recently, a case-report was published regarding a patient who denied approved chemotherapy and instead selected self-administered CBD-oil, which led to the regression of the tumor^[^^17^^]^. Further research (both *in vivo* preclinical studies and clinical trials) is required to evaluate the role of CBD in the management of lung cancer.

## DECLARATIONS

### Ethical statement

Not applicable.

### Data availability

The raw data supporting the conclusions of this article are available from the authors upon reasonable request. 

### Author contributions

VI: conceptualization, formal analysis, investigation, methodology, validation, visualization, writing, and original draft; GPM: formal analysis, investigation, methodology, validation, visualization, writing, and original draft; NZ: Writing, review, editing, and supervising.

### Conflict of interest

None declared.

### Funding/support

Not applicable. 

## Supplementary Materials


